# Ocular Side Effects of Eyelash Extension Use Among Female Students of the University of Benin, Benin City, Edo State, Nigeria

**DOI:** 10.7759/cureus.53047

**Published:** 2024-01-27

**Authors:** Faustina K Idu, Adesuwa D Efosa, Musa Mutali

**Affiliations:** 1 Optometry, University of Benin, Benin, NGA; 2 Optometry, Riant Eye Clinic, Lagos, NGA; 3 Ophthalmology, Africa Eye Laser Centre, Benin, NGA

**Keywords:** tearing, pain, foreign body sensation, redness, eyelids, eyelash extensions

## Abstract

Introduction

Eyelash extensions have become a trendy cosmetic procedure, especially among young female students at various universities. This study assessed the relationship between the usage pattern of eyelash extensions and the ocular side effects arising from eyelash extension use.

Methods

The study was conducted using 400 female undergraduate students at the University of Benin with an age range of 15-30 years and a mean age of 22.50 ± 3.79 years. This observational cross-sectional study was conducted by evaluating the usage pattern of eyelash extensions, ocular side effects, and the relationship between them. Data obtained from the participants were processed using the Pearson correlation analysis in the Statistical Product and Service Solutions (SPSS) (version 22.0; IBM SPSS Statistics for Windows, Armonk, NY) software.

Results

The results showed that only 16% (n*=*64) of the participants had any side effects before fixing eyelash extensions, while 54% (n=216) of the participants had experienced one or more side effects during/after artificial eyelash wear. The most common side effects experienced were itching (n=152, 38%), lashes pulling out (n=144, 36%), heavy eyelids (n=136, 34%), and red eyes (n=136, 34%). Other ocular side effects were grittiness/foreign body sensation (n=128, 32%), tearing (n=96, 24%), burning sensation (n=96, 24%), pain on the eyelids (n=88, 22%), misdirected lashes (n=40,10%), eyelids swelling (n=24, 6%), a boil on eyelids (n=16, 4%), and discharge (n=8, 2%). Moreover, the result showed statistically significant correlation between the usage pattern and the ocular side effects such as pain (r=0.22), itching (r=0.23), tearing (r=0.21), burning sensation (r=0.17), a boil on the eyelids (r=0.21), heavy eyelids (r=0.16), misdirected lashes (r=0.22), red eyes (r=0.15), lashes pulling out (r=0.12), eyelids swelling (r=0.18), grittiness (r=0.10), and discharge (r=0.10) (p<0.05).

Conclusions

It is concluded that there is a relationship between the usage pattern of eyelash extensions and the ocular side effects. Adequate and proper attention should be paid to this ever-growing cosmetic procedure's potential visual health risks.

## Introduction

Eyelashes, also known as cilia, are hairs that grow on the eyelid edges and are fixed to the eyelid by a “root.” They grow in many layers (3-5 layers) on the eyelid border. They are anchored to the eyelid by a “root.” The ectoderm of the human embryo develops into eyelashes between the 22nd and 26th week of pregnancy [1]. There is no need to clip or pluck natural eyelashes as they grow to a specific length and fall out independently.

According to recent observations, eyelash extensions have become a global cosmetic trend because long eyelashes have been associated with femininity in many cultures worldwide. With the help of an adhesive, eyelash extensions made of synthetic fibers (e.g., nylon) or natural fibers (e.g., horsehair, silk, or mink) are individually attached to the natural eyelashes. Individuals with thin or short eyelashes can also benefit from eyelash extensions, typically used with mascara, to produce a "bold effect" or strengthen the natural lashes [2]. Strip lashes, individual flare lashes, and single individual lashes are the three types [3]. During removal, it may irritate the eyelids, clog follicles, and pull out lashes [4].

Karl Nessler, a famous hairstylist and inventor, invented a method to weave artificial eyebrows and eyelashes in the United Kingdom in 1902 (the 20th century) and began selling them at his salon in 1903. In 1911, a Canadian named Anna Taylor received a patent for eyelash extensions in the United States. As procedures developed in the 21st century, artificial facial hair grew more popular with the general population [5]. Eyelash extensions are a widely accepted technique to enhance the appearance of the eyes and face, but they have some drawbacks. The most prevalent of these side effects include chemical conjunctivitis [6], allergies, pain, dermatitis, and conjunctivitis [7].

Eye care practitioners have reported cases where ocular morbidities presented in association with eyelash extensions. It is also well-known that artificial eyelash users frequently complain of side effects ranging from itching to foreign body sensations and pain. The rationale for this study was to assess the relationship between the pattern of usage of artificial eyelash extensions and side effects among the female students of the University of Benin, Benin City.

## Materials and methods

This study was conducted using an observational cross-sectional study design to ensure the proper representation of women within the University Campus. The study population included undergraduate female students of the University of Benin, Benin City. The sample population comprised female undergraduate students aged 15-30 years and a mean age of 22.36 ± 3.79 years. The inclusion criteria were females who had used eyelash extensions for at least six months before the research dates. The sample size was adapted from the study by Abah et al. [2] who used 310 participants; however, the authors increased this number to 400 in this study. Hence, the returned questionnaires were numbered and randomized, and then the first 400 complete forms with a “yes” for eyelash extension use were selected. All participants gave consent to participate in this study.

Self-administered, structured questionnaires were distributed to female students in halls of residences and various faculties to gather data on the demographics of the respondents, their pattern of artificial eyelash use/wear, and the ocular side effects they might have experienced while wearing eyelash extensions. Collection boxes were set up for participants to drop off their filled forms. Information gathered included their demographics, usage pattern, duration, frequency, and types of eyelashes used, whether they reused eyelash extensions, their knowledge of the kind of glue used, method of removal, and where they got their eyelash extensions done. Responses of participants who indicated “yes” to using artificial eyelash extensions were collated and vetted to ensure they fit into the inclusion criteria. Data obtained from the responses were then compiled and sorted for analysis and presentation. Patient demographics and response summaries were presented using tables. A Pearson correlation analysis was used to test the strength of the relationships between side effects and participant's responses. A p value of ≤0.05 was considered significant. Ethical approval was sought and obtained from the ethical committee of the Department of Optometry.

## Results

A total of 1,315 respondents were logged in this study, of which only 400 indicated that they actively used eyelash extensions, giving a response rate of 30.42%. Approximately 16.0% (n=64) noticed some side effects. Out of the 64 (n=16%), 2.0% (n=8) had itching/grittiness, 2.0% (n=8) had itching, heavy eyelids, and tearing, 2.0% (n=8) had itching, tearing, and burning sensation, 2.0% (n=8) had itching, red eyes, and discharge, 2.0% (n=8) had heavy eyelids, and 6.0% (n=24) had tearing.

Out of the 400 participants, 66.0% (n=264) preferred to fix long lashes, while 34.0% (n=136) preferred short lashes. Moreover, 38% (n=152) had fixed eyelashes made from human or horse hair, 24% (n=96) had no idea what type of material their eyelashes were made of, 18% (n=72) had fixed synthetic eyelash extensions, 10% had (n=40) fixed mink eyelash extensions, 8% had (n=32) fixed silk eyelash extension and, lastly, and 2% had (n=8) fixed faux mink eyelash extensions. Therefore, the most preferred type of eyelash material was the human or horse eyelash extension. Approximately 48.0% (n=192) of the respondents used eyelash glue when fixing their eyelash extensions, 38% (n=152) had no idea what type of glue was used in fixing their eyelash extensions, 12% (n=48) used multipurpose bond when fixing their eyelash extensions, and lastly, and 2.0% (n=8) had used other types of glue which they could not specify. The demographics of participants as extracted from questionnaires are shown in Table 1.

**Table 1:** Demographic distribution of the study population

Demographic distribution of the study population		
Frequency	n	%
Monthly	48	12.0
Bimonthly	160	40.0
Quarterly	80	20.0
Occasionally	96	24.0
Total	16	4.0
Who fixed your first eyelash extensions?		
Friend	40	10.0
Local beautician	144	36.0
Professional/expert beautician	216	54.0
Total	400	100.0
What type of glue was used in fixing your eyelash		
Multipurpose bond	48	12.0
Eyelash glue	192	48.0
I don’t know	152	38.0
Others	8	2.0
Total	400	100.0
Reuse of eyelash extensions		
No	200	50.0
Yes	200	50.0
Total	400	100.0
Procedure for removal of artificial lashes		
Pull it out	160	40.0
It comes off on its own	144	36.0
Wash it out	40	10.0
Seeks expert help	56	14.0
Total	400	100.0
How long do you wear your eyelash extensions		
Once per day	40	10.0
Less than once weekly	80	20.0
Once weekly	64	16.0
2 weekly	104	26.0
1 monthly	80	20.0
Total	400	
Any complaints about eyelashes?		
No	184	46.0
Yes	216	54.0
Total	400	100.0

Table 2 shows the number of subjects who experienced the various side effects and those who reported no side effects.

**Table 2:** Ocular side effects experienced after fixing eyelash extensions

Side effects	Rarely		Occasionally		Frequently		Always		Total with side effects		Total without side effects	
	N	%	N	%	N	%	N	%	N	%	N	%
Pain	48	12.0	24	6.0	16	4.0	0	0.0	88	22.0	312	78.0
Itching	48	12.0	48	12.0	48	12.0	8	2.0	152	38	248	62.0
Grittiness/foreign body sensation	16	4.0	96	24.0	0	0.0	16	4.0	128	32.0	272	68.0
Heavy eyelids	80	20.0	32	8.0	8	2.0	16	4.0	136	34.0	264	66.0
Tearing	32	8.0	40	10.0	16	4.0	8	2.0	96	24.0	304	76.0
Red eyes	88	22.0	32	8.0	0	0.0	16	4.0	136	34.0	264	66.0
Discharge	8	2.0	0	0.0	0	0.0	0	0.0	8	2.0	392	98.0
Boil on the eyelids	0	0.0	16	4.0	0	0.0	0	0.0	16	4.0	384	96.0
Misdirected lashes	32	8.0	8	2.0	0	0.0	0	0.0	40	10	360	90.0
Lashes pulling out	88	22.0	40	10.0	8	2.0	8	2.0	144	36.0	256	64.0
Burning sensation	64	16.0	16	4.0	8	2.0	8	2.0	96	24.0	304	76.0
Eyelids swelling	24	6.0	0	0.0	0	0.0	0	0.0	24	6.0	376	94.0

A correlation was conducted to test the relationship between the subjects’ positive responses to three questions and the different side effects. The relationship between subject responses and side effects was tested using a Pearson correlation statistic, as shown in Table 3.

**Table 3:** Relationship between participant responses and side effects *Significant at 0.005; **significant at 0.001

Age			Do you wear eyelash extensions?		Before you had your eyelashes fixed, did you notice any symptoms?		Do you reuse eyelash extensions	
Coefficient		P value	Coefficient	P value	Coefficient	P value	Coefficient	P value
Pain	0.026	0.609	-0.037	0.458	0.390^**^	0.000	0.000	1.000
Itching	0.054	0.279	0.140^**^	0.005	0.393^**^	0.000	-0.069	0.171
Grittiness/foreign body sensation	-0.023	0.642	0.128^*^	0.010	0.138^**^	0.006	0.037	0.462
Heavy eyelids	-0.029	0.566	0.118^*^	0.018	0.092	0.065	-0.060	0.232
Tearing	-0.106^*^	0.033	0.102^*^	0.042	0.469^**^	0.000	-0.166^**^	0.001
Red eyes	-0.062	0.217	0.117^*^	0.019	0.284^**^	0.000	-0.021	0.672
Discharge	-0.146^**^	0.004	0.029	0.561	0.305^**^	0.000	-0.143^**^	0.004
Boil on the eyelids	-0.029	0.567	0.042	0.406	0.170^**^	0.001	.204^**^	0.000
Misdirected lashes	0.048	0.339	0.064	0.200	0.126^*^	0.012	-0.105^*^	0.036
Lashes pulling out	0.030	0.553	0.127^*^	0.011	0.172^**^	0.001	-0.045	0.374
Burning sensation	-0.044	0.384	0.094	0.059	0.163^**^	0.001	-0.024	0.628
Eyelids swelling	0.187^**^	0.000	0.052	0.304	0.101^*^	0.044	-0.084	0.093

## Discussion

In modern societies, considerable emphasis has been placed on the appearance of the eye. From the color of the iris, shape of the eyelids, and even the length of our eyelashes, these characteristics have all been used to distinguish one person from another. The study by Abah et al. similarly suggests that cosmesis is the major reason for eyelash extension use among young females [2].

In this study, it was shown that only 16% (n=64) of the participants had any side effects before fixing eyelash extensions, while 54% (n=216) of the participants had experienced one or more side effects during/after artificial eyelash wear. This confirms the findings by Han et al. who concluded that eyelash extensions had deleterious effects on the ocular surface [8]. The results from Table 2 show that most of the common side effects experienced were itching (n=152, 38%), lashes pulling out (n=144, 36%), heavy eyelids (n=136, 34%), and red eyes (n=136, 34%). This is consistent with findings by Amano et al. who reported keratoconjunctivitis and allergic complaints as the most common side effect [9]. The pathophysiology of these complaints likely proceeds from trauma and hair loss when removing eyelash extensions secured with glue on the eyelids [10]. This results in inflammation of the ocular tissue. Other ocular side effects were: grittiness/foreign body sensation (n=128, 32%), tearing (n=96, 24%), burning sensation (n=96, 24%), pain on the eyelids (n=88, 22%), misdirected natural lashes (n=40,10%), eyelids swelling (n=24, 6%), a boil on the eyelids (n=16, 4%), and discharge (n=8, 2%). These side effects are similar to those in the research participants of Nagendran et al.’s study, where allergies were the most prevalent side effects [11]. Approximately 12% of the respondents reported using multipurpose glue for their eyelashes, and such glues are toxic to the human skin and respiratory system [12-15]. Lindstrom et al. also agreed that the inhalation of eyelash glue can cause occupational asthma [16]. Most participants changed their lashes bimonthly; notably, the severity of symptoms can be affected by how often it is fixed [17].

Pearson's correlation was used to analyze the data to find a relationship between the usage pattern of eyelash extensions and the side effects. The results show that eyelash extension use has a significant relationship with the occurrence of itching (r=0.14, p=0.005), grittiness and foreign body sensation (r=0.128, p=0.01), heavy eyelids (r=0.118, p=0.018), tearing (r=0.102, p=0.042), red eyes (r=0.117, p=0.019), and lashes pulling out (r=0.127, p=0.011). The reuse of eyelash extensions resulted in tearing (r=0.16), discharge (r=0.14), a boil on the eyelids (r=0.20), and misdirected natural lashes (r=0.10) [p≤0.05]. However, it is fair to note that some of these side effects were present before these participants started using eyelashes including ocular pain, itching, grittiness/foreign body sensation, tearing, red eyes, discharge, lashes pulling out, burning sensation, and eyelids swelling [p≤0.05].

The limitations of this study included the submission of incomplete forms by some participants. Moreover, a clinical examination was not conducted on the respondents to rule out other causes of reported symptoms, or if they were present before this study. Our data suggest that despite the procedure's attendant concerns, participants may be unaware of the immediate and potential side effects it may have on their eyes and their ability to see.

## Conclusions

The authors concede that there are several situations where eyelashes are indicated especially in cases of a secondary loss of eyelashes. This study is the first to highlight the potential risks of artificial eyelash extensions on the eye in the southern region of Nigeria. Professional help should be sought when fixing eyelashes to minimize ocular side effects, such as constant itching from the eyelash extensions, as this could result in abrasions and alopecia. Moreover, the loss of lashes caused by eyelash extensions could result in alopecia and so on. Therefore, to reduce the risks of side effects, female clients who use this enhancement should be informed about how eyelash extensions may impair their vision.
